# Gastrointestinal basidiobolomycosis with hepatic dissemination: a case report

**DOI:** 10.1099/jmmcr.0.003269

**Published:** 2014-12-01

**Authors:** Maysaa Abdallah Saeed, Tarig Saleh Al Khuwaitir, Tarek Hamed Attia

**Affiliations:** ^1^​ Faculty of Medicine, Zagazig University, Egypt; ^2^​ King Saud Medical City, Riyadh, Kingdom of Saudi Arabia

**Keywords:** basidiobolomycosis, gastrointestinal infection, zygomycosis

## Abstract

**Introduction:**

Gastrointestinal basidiobolomycosis (GIB) is an emerging fungal infection with a few cases reported worldwide. It is caused by *Basidiobolus ranarum*, which does not usually invade blood vessels and rarely disseminates.

**Case presentation:**

We present a rare case of GIB with hepatic dissemination in a 12-year-old Yemeni boy living in southwestern Saudi Arabia. The initial provisional diagnosis was intestinal lymphoma, and a right hemicolectomy was carried out, but histopathological assessment ruled out lymphoma and suggested intestinal tuberculosis. Two weeks after starting antituberculous medications, the patient was referred to our hospital because of fever and right upper abdominal discomfort. There was leukocytosis with marked eosinophilia, and a liver biopsy showed evidence of *B. ranarum* infection. A second opinion by histopathological examination of resected tissues diagnosed colonic basidiobolomycosis. The patient was treated successfully with itraconazole.

**Conclusion:**

GIB is an emerging disease in southwestern Saudi Arabia and should be considered in a patient with an abdominal mass and eosinophilia coming from this region. Persistent elevation of leukocytes and eosinophils after surgical resection of the affected tissue could be used as a predictor of fungal dissemination. Further research is needed for a better understanding of GIB.

## Introduction

Basidiobolomycosis is a rare fungal infection caused by *Basidiobolus ranarum*, an environmental saprophyte, which is a member of the order Entemophthorales, class Zygomycetes (De Leon–Bojorge *et al*., 1988; Kwon–Chung & Bennett, 1992). *B. ranarum* is found worldwide in soil, decaying organic matter and the gastrointestinal tracts of amphibians, fish, reptiles and insectivorous bats (Gugnani, 1999). Basidiobolomycosis is usually a subcutaneous infection, involving the limbs, trunk or buttocks, and mostly affects young healthy males (Sugar, 2005). Most cases have been reported from Africa, South America and tropical Asia (Gugnani, 1999).

The visceral form of infection is rare (Kwon–Chung & Bennett, 1992). Gastrointestinal basidiobolomycosis (GIB) has rarely been reported and poses diagnostic difficulties as its clinical presentation is non-specific, with no identifiable risk factors, all age groups are susceptible and victims of this infection are usually healthy immunocompetent subjects (Lyon *et al*., 2001; Nemenqani *et al*., 2009; Yusuf *et al*., 2003). *B. ranarum* does not usually invade blood vessels and rarely disseminates (Pasha *et al*., 1997).

Here, we report a rare case of GIB, which disseminated to involve the liver, in a Yemeni patient who was treated successfully.

## Case report

A 12-year-old Yemeni boy, living in Abha, Aseer province, Kingdom of Saudi Arabia, was referred with a 2-month history of diffuse abdominal pain, non-bilious vomiting, poor appetite and weight loss. There was no fever, diarrhoea, constipation or jaundice. He did not have melena or rectal bleeding. There was no pertinent history with regard to the child’s outdoor activities.

Apart from a high erythrocyte sedimentation rate (ESR) of 90 mm h^−1^ and leukocytosis without eosinophilia, his laboratory results were within normal ranges. Abdominal computerized tomography (CT) showed a markedly thickened oedematous wall with stratification of the ascending colon including hepatic flexure with an adjacent part of the transverse colon as well as the caecum and the terminal ileum. A large pericaecal mass of 7×4 cm and multiple enlarged regional lymph nodes were noted. An exploratory laparotomy revealed a mass adhering to the anterior abdominal wall, involving the caecum, ileum and ascending colon, with multiple mesenteric lymph nodes. A right hemicolectomy was carried out with ileocolic anastomosis and resection of the mesenteric lymph nodes, as the provisional diagnosis was lymphoma. Histopathological assessment of the resected material ruled out lymphoma and suggested intestinal tuberculosis. Antituberculous medication was started immediately and the patient’s complaints disappeared.

Two weeks after the relief of symptoms, he was referred to our hospital (King Saud Medical City) because of fever and right upper abdominal discomfort.

**Fig. 1.**
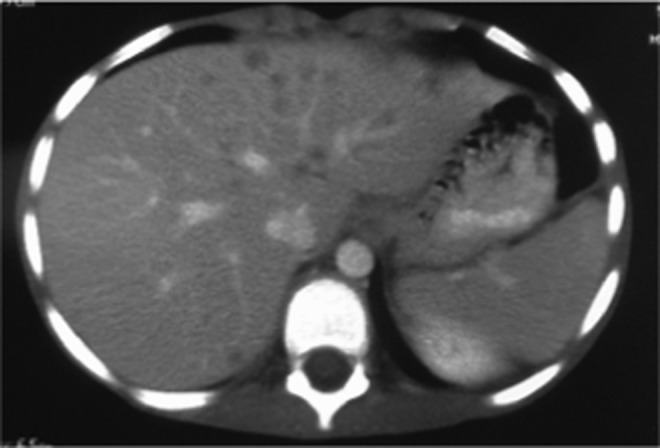
Abdominal CT showing multiple small, low-attenuation lesions scattered throughout both lobes of the liver, some showing ring enhancement.

**Fig. 2.**
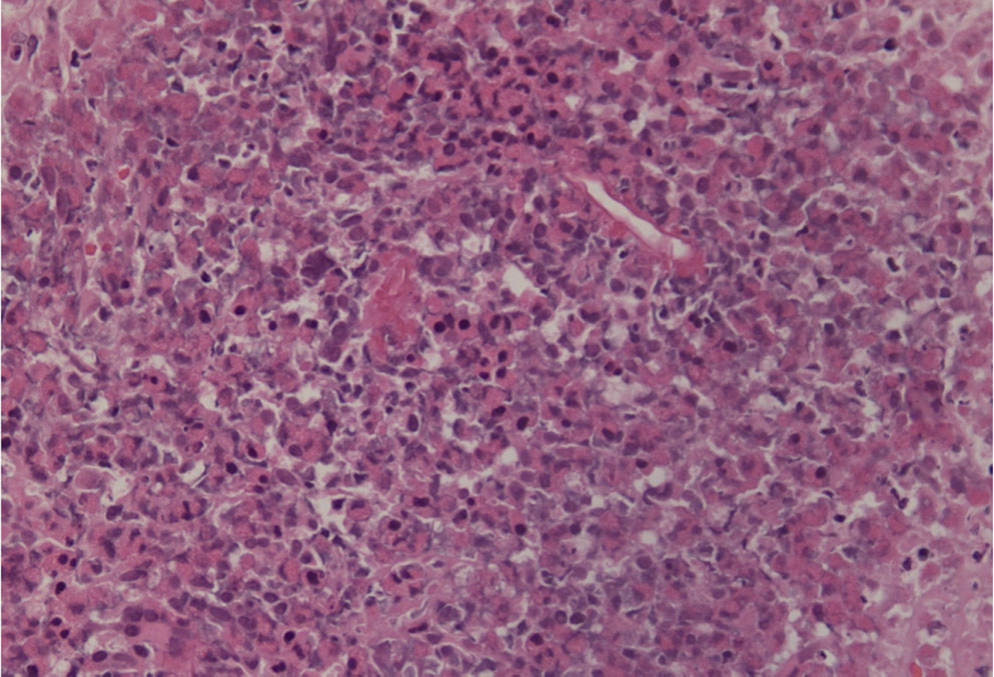
Hepatic *B. ranarum* appearing as broad aseptate fungal hyphae surrounded by an eosinophilic sheath. Haematoxylin and eosin stain. Original magnification ×40.

**Fig. 3.**
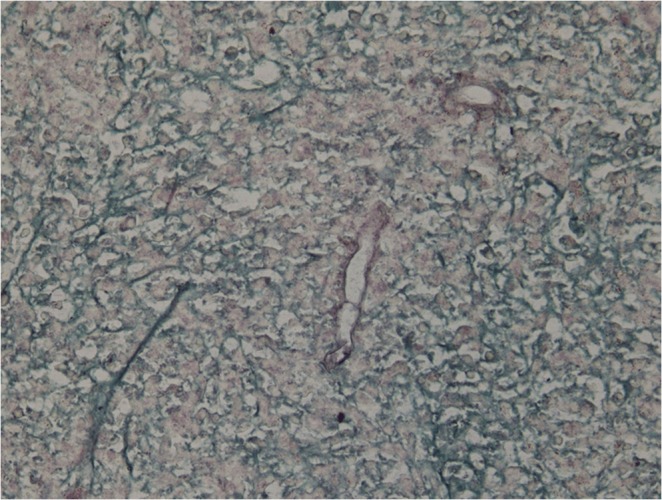
Hepatic *B. ranarum* appearing as broad aseptate fungal hyphae. Grimelius methenamine silver stain. Original magnification ×40.

On examination, he looked unwell and emaciated, his temperature was 39.5 °C and he had tenderness in the right upper quadrant of the abdomen. His laboratory work-up showed the following: white blood cells (WBCs) 19 000 mm^−3^, eosinophils 23 %, haemoglobin 9 g/dl, platelets 4×10^5^ mm^−3^, ESR 120 mm/h and C-reactive protein 90 mg/dl, with normal liver and renal profiles. An abdominal CT revealed multiple small, low-attenuation lesions scattered throughout both hepatic lobes, some showing ring enhancement, an appearance highly suggestive of liver abscess (Fig. 1). A percutaneous liver biopsy was done under ultrasound guidance and sent for histopathological evaluation, which revealed pyogranulomatous lesions rich in eosinophils with broad aseptate fungal hyphae surrounded by eosinophilic material (Splendore–Hoeppli phenomenon). A Grimelius methenamine silver stain of the liver specimen also showed broad aseptate fungal hyphae consistence with *B. ranarum*. (Figs 2 and 3). A molecular diagnosis was not available in the hospital.

Itraconazole treatment was started immediately at a dose of 100 mg twice daily. The itraconazole trough level was checked after 1 week of therapy to confirm its absorption and was >1.0 µg/ml. The general condition of the patient improved after 1 week and he was discharged to be followed up at the outpatient clinic. Seven days after initiation of itraconazole therapy, the patient’s complete blood count showed total WBCs of 11 000 mm^−3^ with 10 % eosinophils. After 1 month, the WBC count was 9900 mm^−3^ with 1 % eosinophils.

**Fig. 4.**
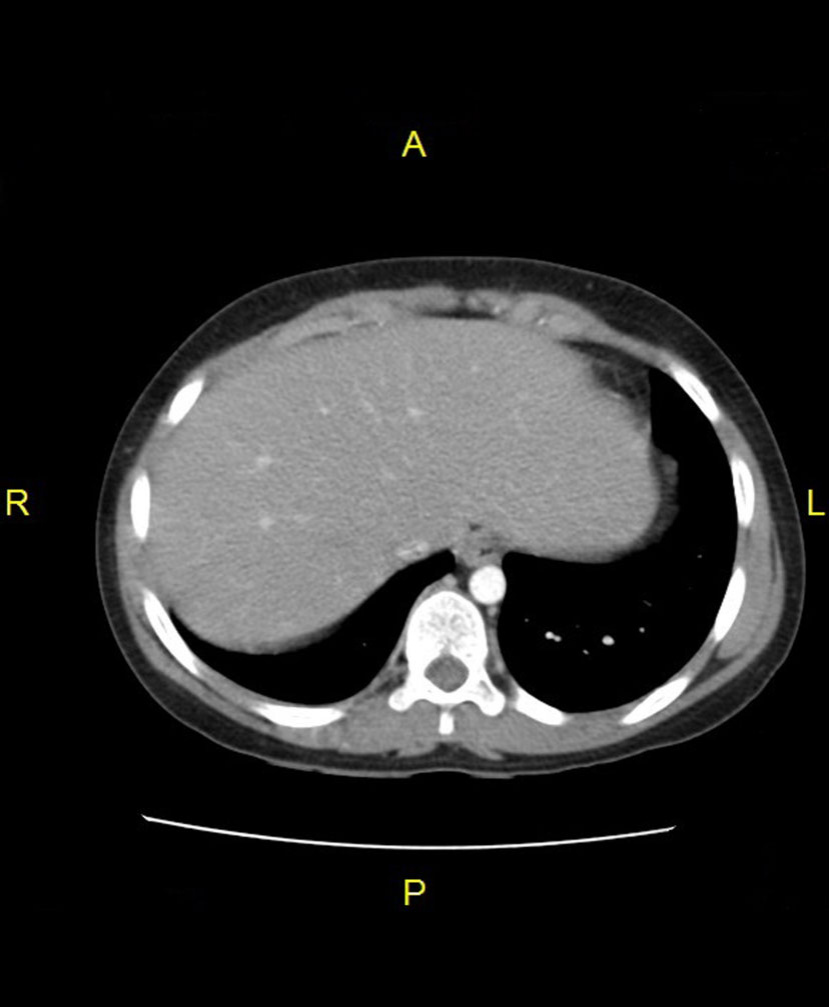
Liver CT after treatment.

After complete clinical and radiological improvement (Fig. 4), itraconazole was discontinued after 12 months of treatment. A second opinion by histopathological examination of the resected material diagnosed *B. ranarum* infection.

## Discussion

Basidiobolomycosis is an unusual fungal infection that manifest in the skin and rarely involves other system. It is caused by *B. ranarum*, which causes infection in immunocompromised hosts and is an opportunistic pathogen in immunocompetent hosts (Khan *et al*., 2001). GIB has rarely been reported in the medical literature although sporadic cases have been reported worldwide (Vikram *et al*., 2012). To our knowledge, 50 cases of GIB have been reported worldwide in addition to our current report (Al Jarie *et al*., 2003, 2011; Al-Qahtani *et al*., 2013; Al Saleem *et al*., 2013; Bigliazzi *et al*., 2004; Lyon *et al*., 2001; Nemenqani *et al*., 2009; Saadah *et al*., 2012; Vikram *et al*., 2012). Most of these cases were reported from tropical areas where the climate was warm and humid, and such a climate enhances the growth of the fungus, suggesting environmental contamination (Al Jarie *et al*., 2003). Al-Qahtani *et al*. (2013) reported three cases of GIB, living in the same area as our patient, Aseer province in southwestern Saudi Arabia. They concluded that GIB is an emerging disease in Saudi Arabia.

No specific risk factors for GIB have been identified; however, prior ranitidine use and longer residence in high-risk areas of endemicity may contribute to the risk (Lyon *et al*., 2001; Khan *et al*., 2001). It is unclear how the fungus is introduced into the host’s gastrointestinal tract, but this probably occurs through ingestion of contaminated soil, animal faeces or food (Fahimzad *et al*., 2006). We could not detect any information about the method by which our patient acquired the infection.

The clinical manifestations of GIB include abdominal pain, fever, vomiting, weight loss and abdominal mass; the latter is observed during abdominal examination or is demonstrated by imaging or during laparotomy (Vikram *et al*., 2012). The presence of an abdominal mass may be confused with malignancy, appendicular mass, Crohn’s disease, intestinal tuberculosis, sarcoidosis and amoebiasis (Ribes *et al*., 2000). However, the characteristic histopathological features are the key in the diagnosis of all cases, together with fungal culture in some cases (Al-Qahtani *et al*., 2013; El–Shabrawi *et al*., 2011). This means that in cases where there is a lack of awareness and the facilities to diagnose such a rare disease, the diagnosis will be missed and such patients are at increased risk of morbidity.

Leukocytosis, marked eosinophilia and elevated ESR and C-reactive protein were found in our case, as in other reports (Al-Qahtani *et al*., 2013; Al Saleem *et al*., 2013). These laboratory results should alert the physician to suspect GIB in the presence of clinical presentations suggestive of the disease. Lyon *et al*. (2001) mentioned in his report that leukocytosis resolved after surgical resection. Our patient had markedly elevated leukocytic and eosinophilic counts after hemicolectomy. This elevation can be explained by fungal dissemination to the liver, so persistent elevation of leukocytic and eosinophilic counts could be assumed to be predictors of fungal dissemination.

The characteristic histopathological features of GIB include transmural granulomatous inflammation composed of abundant eosinophils, lymphocytes, histiocytes and giant cells. Histochemical stains revealed broad, aseptate hypha-like structures surrounded by an eosinophilic sheath (Splendore–Hoeppli phenomenon) (Hussein *et al*., 2007).

*B. ranarum* generally does not invade blood vessels and rarely disseminates (Pasha *et al*., 1997). However, van den Berk *et al*. (2006) presented a case of an obstructing colon tumour with associated liver mass, whilst Bigliazzi *et al*. (2004) presented a case of disseminated basidiobolomycosis in an immunocompetent woman, with lung involvement as the first clinical manifestation. The prognosis of GIB is usually favourable; however, both of these cases had a fatal outcome. Our patient initially presented with colonic basidiobolomycosis, which disseminated to involve the liver, and was cured.

The appropriate treatment of GIB has not been outlined precisely. Based on the available information, it appears that optimal treatment is combined surgical treatment and prolonged use of antifungals (Fahimzad *et al*., 2006).

However, Al Saleem *et al*. (2013) avoided surgical intervention and their patient showed an excellent response to oral voriconazole. They mentioned in their report that whether surgery is needed or not depends on the nature of the disease and its location, extension and the patient’s condition. The best choice of antifungal agent is not clear, but itraconazole has been used with success in many reports (Al Jarie *et al*., 2003; Fahimzad *et al*., 2006). Clinical failure has been described with amphotericin B. Potassium iodide has been used with success in the treatment of subcutaneous basidiobolomycosis (Vikram *et al*., 2012); however, Nazir *et al*. (1997) reported a case of invasive retroperitoneal infection with no response to potassium iodide. A hemicolectomy was carried out for our patient before reaching the right diagnosis, and hepatic involvement was successfully treated with itraconazole.

## Conclusion

GIB is an emerging disease in southwestern Saudi Arabia, so special attention should be given to patients with an abdominal mass and eosinophilia coming from this region. Persistent elevation of leukocytes and eosinophils after surgical resection of the affected tissue can be used as a predictor of fungal dissemination. Further research is needed for a better understanding of GIB.

## Abbreviations

CT: computed tomography
ESR: erythrocyte sedimentation rate
WBC: white blood cell.
